# Contraception Use Among Iranian Women With HIV: A Qualitative Study

**DOI:** 10.5539/gjhs.v8n1p199

**Published:** 2015-05-15

**Authors:** Sara E. Saeieh, Alireza N. Nasrabadi, Abbas Ebadi, Zahra B. Moghadam, Minoo Mohraz, Zahra B. Jozani, Elham Rezaei

**Affiliations:** 1 School of Nursing and Midwifery, Tehran University of Medical Sciences, Tehran, Iran; 2 Behavioral Sciences Research Center, Nursing Faculty of Baqiyatallah University of Medical Sciences, Tehran, Iran; 3 Iranian Research Center of HIV/AIDS, Iranian Institute for Reduction of High-Risk Behaviors, Tehran University of Medical Sciences, Tehran, Iran; 4 Department of Midwifery, Nursing and Midwifery Faculty, Urmia University of Medical Sciences, Urmia, Iran

**Keywords:** contraception, HIV, women, qualitative, study

## Abstract

**Background:**

The application of family planning methods to people with HIV not only prevents unwanted pregnancy, but also leads to a reduction in the possibility of transmission of the virus from the patient to the sexual partner and the fetus. In order to prevent the spread of HIV and enhance reproductive rights, it is necessary to inform women with HIV of the contraception methods.

**Objective:**

The aim of this study was to explore experiences of HIV positive women about contraception use.

**Method:**

This qualitative study was conducted on 18 women with HIV who were at reproductive age and had referred the Center for clients with Risky Behavior in Imam Khomeini Hospital. Data were analyzed using the conventional content analysis method in MAXQDA 10.

**Results:**

The following two themes were derived from descriptions of the use of contraception methods by women with HIV: 1) Contraception is the forgotten component of reproductive health services; 2) inconsistent condom use. Each theme also contained three sub-themes.

**Conclusion:**

Results of investigations showed that Risky Behavior consultation Centers mostly stress the use of condom for husband/sexual partners without HIV. In addition, since health care practitioners play an important role in provision of reproductive health services, their lack of knowledge and cooperation considerably contribute to the spread of the disease and violation of patient rights.

## 1. Introduction

Today, 34 million patients with HIV are living in the world. Of this number, about 40% of female at reproductive age (HIV/AIDS, 2012). According to the latest published statistics, the number of people with HIV in Iran is 27888, but authorities believe that this figure has to be multiplied by three to include people who are suffering from HIV but are not aware of their disease. 88.7% of the patients are men and 11.3% are women. Most of these women are of reproductive age and are sexually active (HIV news, 2014). If people with HIV use family planning methods, they not only can avoid unwanted pregnancy but also can reduce the risk of transmission of the virus to their sexual partner or fetus. In addition, it is a more cost-effective means of preventing the transmission of this virus (Halperin, Stover, & Reynolds, 2009).

The use of contraception methods is an important index in the population’s conscious family planning attempts (Kaida, Laher, Janssen, & Money, 2011; Smith, Ashford, Gribble, & Clifton, 2009).

It is recommended to provide consultation services to all women with HIV on the use of the dual contraception methods (condom with another contraception method) (CDC, 2012). Unfortunately, in spite of the clear relationship between family planning and AIDS, these two are rarely integrated (Wilcher & Cates, 2010). Reproductive health services can effectively support women with HIV in benefiting from their reproductive rights (e.g. deciding on the number of children and spacing children or timing) (WHO, 2006).

If women with HIV have effective access to contraception methods, it will be possible to enhance their pregnancy rights and attain the following three goals of the Millennium Development Goals (MDG): reducing child mortality, enhancing the health of the mother, and preventing the spread of AIDS. However, reports from all over the world still suggest that the needs of women with HIV for contraception methods are not met and they are experiencing unwanted pregnancies (Adair, 2009; Harrington et al., 2012; Schwartz et al., 2012).

The majority of HIV prevention programs running in Iran are focused on injection drug users (IDUs) and transmission of AIDS through infected syringes. However, with the beginning of the third wave of prevalence of HIV in Iran, the rate of transmission of HIV among women has increased. Currently, there is increased concern for transmission of HIV through sexual intercourse (CDC, 2011).

Hence, it seems necessary to gather information on HIV female patients’ knowledge of the use condoms for sexual intercourse (as the most effective way of preventing the transmission of HIV to sexual partners) and the use of contraception methods for prevention of unwanted pregnancy and birth of babies with HIV in the cultural context of Iran. Since little information was available on the use of contraception methods by Iranian women with HIV, and since no study was previously conducted on the experiences of women with HIV about contraception methods, this qualitative study aimed to explore these experiences.

## 2. Method

This was a qualitative study, the first author (PhD student) conducted in-depth, semi-structured interviews. The intent of the interviews was to obtain narratives describing how participants had experienced contraception use. Participants shared their understanding of situations about contraception use that they had countered during their infection with HIV.

### 2.1 Sample/Participant

This study included 18 in- depth interviews taken from women with HIV in the age of fertility who were referred to Imam Khomeini Hospital Consultation Center for clients with risky behaviors in Tehran, Iran. Data collection started in November 2013 and ended in March 2014. These women were selected in consultation with midwife of behavioral Counselling Centre.

### 2.2 Data Collection

The data were collected by semi-structured, open-ended interviews conducted by the first author. She is PhD student and faculty member of university and she had experiences about qualitative study and she was supervised of clinical course of students in risky behavior center. Participants were selected by purposeful sampling with maximum variation. Initially, first interview was conducted by first and corresponding authors, after approval by the other members of the research team next interviews were conducted. Interviews continued until saturation of the data was reached and the researcher was no longer obtaining any new data. All women were interviewed in a private room in the positive club in the Iranian Research Center for HIV. Only interviewer and participant present in place of interview. Interviews with women with HIV took from 45–90 min on average. An interview questions was developed during interview and research team opinions (Table 1). Interviews were tape recorded with permission. The interviewer took field notes immediately after each interview. And also during interview each participant response to question about age, education, marital status, job, and husband/partner HIV status, children, time of diagnosis, transmission, family income.

**Table 1:** Sample of interview questions

1. Can you please tell me about your diagnosis experience? 2. Please explained about intention of fertility? 3. Please describe experiences about contraception use? 4. Please describe experiences about condom use?

### 2.3 Data Analysis

Data analysis was performed on guide by Graneheim and Lundman (Graneheim & Lundman, 2004), the following steps were taken to analyze the collected data:

1- Transcribing the interviews verbatim and reading through several times to get a general sense of the material.
2- Dividing the text into meaning units, which are key phrases in the text.
3- Abstracting the condensed meaning units and outlining with codes.
4- Grouping codes into sub-categories and categories based on comparisons regarding their similarities and differences.
5- Re-organizing and merging into sub-themes and overarching themes. Data were analyzed with MaxQDA 10software. The authors worked as a team throughout this process, the first author doing the initial analysis and the corresponding author, second and third authors (faculty members) reviewing the analysis during the various stages of the analysis process. In second stage of analysis for more accuracy, first author and corresponding author coding the meaning units separately and compared codes together. The sixth authors discussed the analysis together to resolve any problems.

### 2.4 Rigor

In this study, various aspects of trustworthiness have been observed. Credibility was established through member checking, peer checking, and prolonged engagement. Member checking was done by asking the respondents to approve the transcripts and emerging codes from the interviews. Research teams consulted with each other to deal with any ambiguities in the coding process, categories and themes. In areas where the two researchers did not agree, definitions were clarified and discussion continued until consensus was reached. For addressing transferability, the complete set of data analysis documents are on file and available upon request.

### 2.5 Ethical Considerations

This study is one part of the author’s doctoral dissertation. The Ethics Committee of Tehran University of Medical Sciences approved the study proposal and corroborated its ethical considerations. All participants were informed about the purposes and the methods of this study. Before interviews, all respondent were signed informed consent. They were informed that they had a right to refuse to participate in the interview and this right could be exercised at any time without having any negative impact on the services delivered to them. The permission to type record the interviews was obtained from the participants.

## 3. Results

Participant demographic information is summarized in Table 2.

**Table 2:** Characteristics of the study population

Characteristic	Number	percent
Age		
22-27	7	38.8
28-33	5	27.7
34-39	5	27.7
40-45	1	5.5

Marital status		
Single	2	11.11
married	13	72.2
divorced	3	16.6

Education		
Primary or less	3	16.6
Secondary	5	27.7
Diploma	8	44.4
University	2	11.11

Job		
House keeper	12	66.6
employee	3	16.6
Informal job	3	16.6

Transmission		
Out of marriage sexual relationship	3	16.6
Infected husband	12	66.6
Unknown	3	16.6

Time of diagnosis		
<1 years	2	11.1
1-5 years	10	55.6
6-10 years	4	22.2
≥11	2	11.1

Partner / husband status		
Negative	5	27.7
Positive	10	55.6
Unknown	3	16.6

Currently living children		
0	6	33.3
1	7	38.8
2	4	22.2
3	1	5.5

Having infected child		
0	13	72.2
1	5	27.7

Family income		
Good	4	22.2
Average	9	50
Bad	5	27.8

Fertility desire		
Yes	4	22.2
No	14	77.8

The following two themes were derived from descriptions of the use of condoms and contraception methods by women with HIV: 1) Contraception is the forgotten component of reproductive health; this them include three subtheme: i) Shortage of Counselling Contraception Methods of Women with HIV, ii) Unavailability of Some Contraception methods in Centers for clients with risky behavior, iii) Possibility of Legal Abortion.

Another theme was 2) inconsistent condom use. Each theme also contained three sub-themes, this theme and also contained three sub themes: i) The Effect of Man’s Power ii) Belief in the Ineffectiveness of Condom iii) Disclosure of HIV Status.

### 3.1 Contraception: The Forgotten Component of Reproductive Health

The use of contraception methods by patients with HIV is one of the most effective ways of preventing the spread of HIV. However, the rate of contraception use was very low among participants.

#### 3.1.1 Shortage of Contraception Counselling

According to the results of this study, only one of the participants was using a contraception method other than condom following the transmission. Most participants were not informed about other contraception methods and therefore lacked adequate information on the dual use of contraception methods. Fear of side effects and a lack of adequate information were other causes of the reduction in the use of contraception methods. The concern for effects and the fear of interference of contraception methods with the disease were also among other reasons, for refusing/stopping the use of contraception methods

One of the participants, who had experienced unwanted pregnancy and had given birth to a child with HIV, said: “Well, they only gave us condom and did not provide us with any other information. I also did not think I would become pregnant due to my disease. I was only thinking about my disease and protecting my two daughters against this disease. I didn’t think that I became pregnant again. So I became pregnant with this child, who has the disease as well.” (Participant, No. 8)

#### 3.1.2 Unavailability of Some Contraception Methods in Centers for Clients With Risky Behavior

Some contraception methods have not provided to those who visit the related centers and the unavailability of these methods is another obstacle in the use of these methods. Many people visit these centers from distant cities and places to receive the services. Therefore, it is easier for them to receive family planning services and other services in centers for clients with risky behavior.

One of the participants, who did not have a child and was not interested in having children, said: “I use a condom but I am always afraid of pregnancy. I tried to take pills several times, but the pill gave me sickness. I also used to forget if I had taken the pills or not. They told me to take the ampoule, but when I came here they told me they don’t have ampoules and I have to either use condom or take pills. They told me to take the tubal ligation surgery. But I’m afraid of surgery. I don’t have any children yet and I don’t want any. I became pregnant two times and I aborted the fetus. I don’t want to go through tubal ligation surgery as well. So I have a stressful intercourse with my husband.” (Participant, No. 13)

Another participant said: “You know, not every man likes condoms! You cannot also insist on using them. I was teaching illiterate sex worker women when I realized they were receiving condoms for women. So if the man refuses to use condoms, the women can. But they don’t provide those condoms here.” (Participant, No. 14)

#### 3.1.3 Possibility of Legal Abortion

In Iran, women with HIV are legally authorized to abort their fetus and there is no problem in this regard. Most of the participants had adequate information in this regard. However, due to the possibility of this option, they lower their use of contraception methods and consider abortion a contraception method.

One of the participants, who had a child with HIV and were not willing to have another child, said: “I want to have children, but since I am sick I don’t want any other child unless there is a cure for this disease. A few days ago I saw the news on the TV about a vaccine for AIDS as well as drugs that are produced for AIDS. They are testing the methods. Besides, we have little intercourse and I use natural prevention methods. If I become pregnant, I will abort it. I have once visited here and I have once obtained a certificate to abort it.” (Participant, No. 9)

### 3.2 Inconsistent Condom Use

The most important way of preventing the spread of AIDS is using condoms in sexual relationships. Only two of the participants, whose husbands/sexual partner were not infected with AIDS and were aware of the disease, were constantly using condoms. This theme includes the following three subthemes: 1) the effect of man’s power, 2) belief in the ineffectiveness of condom, 3) lack of disclosure of HIV status.

#### 3.2.1 The Effect of Man’s Power

Man’s interest is one of the important factors contributing to the use of condom in sexual relationships. Many participants referred to the unwillingness of their mate or sexual partner to use condom and many of them mentioned that they could not insist on the use of condom because they could be subject to the violence practiced by their mate/sexual partner.

One of the participants who had one child said: “My husband doesn’t like to use condoms. He says condom ruins the joy of sex. So I insist on it, he will start a quarrel and says ‘you are sick, what’s difference for you?” (Participant, No. 12)

#### 3.2.2 Belief in the Ineffectiveness of Condom

Some of the participants mentioned that they had an experience with torn condoms and pregnancy and therefore considered condom to be useless due to the possibility of its failure as well as the possibility of its rupture.

One of the participants who was divorced and who had caught AIDS through a non-marriage relationship said: “I became familiar with a man who didn’t know he was infected. He was informed following tests. They told me in this center that it was not possible for me to catch this disease if I used condoms. But the whole condom concept is ridiculous to me now. I tore condoms two times and the second time I visited the center late and I couldn’t consume the drugs on time. So I am infected with this disease two. So I don’t want to be with someone who doesn’t have AIDS because I caught it through sexual relationships and so I think there is a high possibility of rupture” (Participant, No. 11)

#### 3.2.3 Disclosure of HIV Status

Disclosure of HIV status to one’s sexual partner is the responsibility of every human being with AIDS, who has to be trained on this. It is also among the most effective ways of preventing the spread of the disease. In this research, three participants with non-marriage sexual relationships said they were unwilling to disclose their HIV status to their sexual partners. Therefore, one of the reasons for discontinuous use of condoms in sexual relationships was the lack of awareness of sexual partners about the HIV status of their partners.

One of the participants said: “I won’t tell I’m sick because if I do my partner will leave me. The one who gave me this disease hadn’t tell me as well! I have not told my current partner because you can’t say who gives it to you. I ask him to use condoms because I can take contraception pills, but sometimes he doesn’t like to use it and I don’t insist as well because otherwise he may become suspicious and leave me.” (Participant, No. 17)

## 4. Discussion

Results of this study indicated that the use of contraception methods other than condoms has reduced among women with HIV. Although the WHO approves all of the contraception methods for women with HIV, results of research on women with HIV indicate that a few number of women with HIV use contraception methods as compared to the general public (WHO, 2012). There are few studies on the effect of HIV on the use of contraception methods and the existing studies present varying results. Some of the studies reported that there is a positive relationship between HIV infection and the use contraception methods. For example, in Zimbabwe women with HV are more interested in the use of these methods compared to healthy women (Elul et al., 2009).

Another study in Kenya and Zambia also showed that women with and without HIV use contraception methods equally (Rutenberg & Baek, 2005).

Fear of the side effects of contraception methods, which is the result of previous experiences of the person and others and concerns for health threats among people with HIV (including the concern for the interference between hormonal methods and HIV or anti-retroviral drugs) as a result of lack of accurate information on this issue (Laher et al., 2009).

Unavailability of all contraception methods in centers for risky behavior disorders is considered, in this research, to be another factor contributing to the reduction in the use of such methods. Access to family planning services in HIV centers leads to an increase in the use of non-condom contraception methods as well as dual protection in women. With provision of such an access the quality of services is enhanced, customer satisfaction is increased, and the relationship between clients and services provides operating in reproductive and sexual health centers grows (Spaulding et al., 2009). In order to add to the means of contraception and prevention of sexually transmitted diseases (STD) for women with HIV, it seems necessary to provide them with female condoms (Kendall, 2013).

Results of this study revealed that except for participants with healthy husbands (HIV negative), other participants were not constantly using condoms. Various studies also revealed that one quarter to three quarters of women with HIV were not constantly using condoms for sexual intercourse (Ayiga, 2012; Chakrapani, Newman, Shunmugam, & Dubrow, 2010; Walusaga, Kyohangirwe, & Wagner, 2012). Obstacles in the way of using condoms in this research were the unwillingness of males to use condom (due to a reduction in sexual desire), the belief in ineffectiveness of condom, and lack of disclosure of HIV status (as a result of a lack of responsiveness) to one’s sexual partner. Other studies also referred to the following obstacles in the use of condoms: boredom with condoms, lack of interest in protected sexual relationship, men’s resistance to condom, and the effects of gender inequality (such as the economic dependence of women to men and violence against women) (HIV, 2012; Lang, Salazar, Wingood, DiClemente, & Mikhail, 2007; Ngure et al., 2012; Raiford, Wingood, & DiClemente, 2007).

As it was reported by other studies, men’s gender power is an important factor influencing the use of condom and spread of AIDS. Most women cannot insist on the use of condom as they are afraid of their demand or the sexual desire of their men (Jewkes, Levin, & Penn-Kekana, 2003). In order to prevent the transmission of HIV, it is necessary to spread the notion of positive prevention among people with HIV. There is no single definition of positive prevention, but it generally refers to action taken by HIV patients to protect their health and avoid transmission of HIV to others (Kennedy, Medley, Sweat, & O’Reilly, 2010).

Another factor that was considered to influence the continuous use of condoms was a lack of disclosure of HIV status to sexual partners. A study in Uganda showed that female HIV patients who had not disclosed their HIV status to their sexual partners used condom at a lower rate (Wanyenze et al., 2011). People who constantly disclose their HIV status are less interested in showing unsafe behavior. The refusal for disclosing HIV status results from the fear of getting rejected by one’s sexual partner. In addition, it is also possible for the person refusing to do so to lose the economic support of his sexual partner in case she reveals the status (Stutterheim et al., 2011; Visser, Neufeld, De Villiers, Makin, & Forsyth, 2008).

Healthcare providers play an important role in the provision of reproductive health. Therefore, their lack of information or refusal to fulfill their obligations can considerably contribute to the spread of the disease and violation of patient rights. Some of the factors contributing to the improvement of reproductive rights of women with HIV and the reduction in the spread of this disease are as follows: teaching healthcare providers on this disease; integrating reproductive health services in AIDS centers to present all contraception methods; provision of proper consultation; and controlling services provided by these centers.

## 5. Conclusion

Results of this study reflect the necessity of integrating reproductive health services and HIV/AIDS. Since men’s participation is one of the principles of reproductive health services and since men reportedly play an important role in the use of condom and other contraception, it is recommended to provide consultation to couples. Consequently, men’s participation will be increased. In addition, it is recommended to make interventions to increase individual constructive skills and enable people to disclose their HIV status, especially to their sexual partner. Moreover, it is also recommended to hold group support sessions and consultation programs on disclosure of diseases to increase the self-confidence of patients. As mentioned, provision of proper consultation and accurate information on family planning services and reproductive health, use of dual birth control and consistent use of condoms shall be prioritized in center for clients with risky behavior.
